# Selective inhibition of arginase-2 in endothelial cells but not proximal tubules reduces renal fibrosis

**DOI:** 10.1172/jci.insight.142187

**Published:** 2020-10-02

**Authors:** Michael D. Wetzel, Kristen Stanley, Wei Wei Wang, Soumya Maity, Muniswamy Madesh, W. Brian Reeves, Alaa S. Awad

**Affiliations:** Department of Medicine, University of Texas Health Science Center at San Antonio, San Antonio, Texas, USA.

**Keywords:** Nephrology, Chronic kidney disease, Mitochondria, Nitric oxide

## Abstract

Fibrosis is the final common pathway in the pathophysiology of most forms of chronic kidney disease (CKD). As treatment of renal fibrosis still remains largely supportive, a refined understanding of the cellular and molecular mechanisms of kidney fibrosis and the development of novel compounds are urgently needed. Whether arginases play a role in the development of fibrosis in CKD is unclear. We hypothesized that endothelial arginase-2 (*Arg2*) promotes the development of kidney fibrosis induced by unilateral ureteral obstruction (UUO). Arg2 expression and arginase activity significantly increased following renal fibrosis. Pharmacologic blockade or genetic deficiency of *Arg2* conferred kidney protection following renal fibrosis, as reflected by a reduction in kidney interstitial fibrosis and fibrotic markers. Selective deletion of *Arg2* in endothelial cells (*Tie2^Cre^/Arg2^fl/fl^*) reduced the level of fibrosis after UUO. In contrast, selective deletion of *Arg2* specifically in proximal tubular cells (*Ggt1^Cre^/Arg2^fl/fl^*) failed to reduce renal fibrosis after UUO. Furthermore, arginase inhibition restored kidney nitric oxide (NO) levels, oxidative stress, and mitochondrial function following UUO. These findings indicate that endothelial Arg2 plays a major role in renal fibrosis via its action on NO and mitochondrial function. Blocking Arg2 activity or expression could be a novel therapeutic approach for prevention of CKD.

## Abstract 2

Endothelial-arginase-2 plays a major role in renal fibrosis via its action on nitric oxide and mitochondrial function.

## Graphical abstract

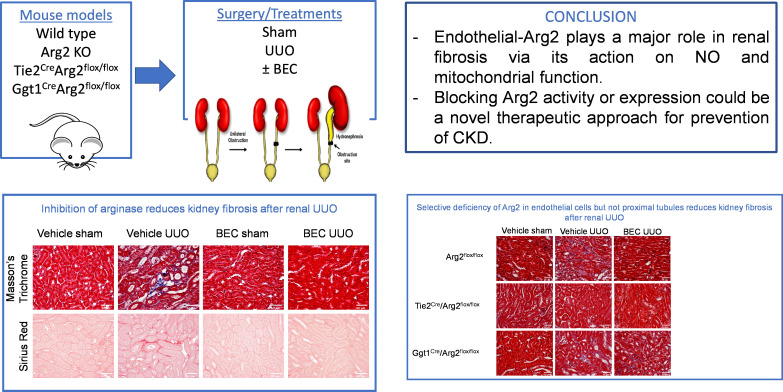

## Introduction

Novel therapeutic interventions for preventing or attenuating renal fibrosis remain a focus of significant interest. Fibrosis is the final common pathway in the pathophysiology of most forms of chronic kidney disease (CKD); it involves glomerular sclerosis and/or interstitial fibrosis that ultimately lead to end-stage renal failure (1–3). A major hallmark of renal tubulointerstitial fibrosis is the accumulation of myofibroblasts and extracellular matrix proteins (4–7). Unilateral ureteral obstruction (UUO) is an established experimental model of progressive renal tubulointerstitial fibrosis (8). Ureteral obstruction results in marked renal hemodynamic and metabolic changes, followed by tubular injury and cell death by apoptosis or necrosis, with interstitial macrophage infiltration (9). Currently, no effective interventions are available to alleviate renal fibrosis in clinical practice. Therefore, exploring the molecular mechanism of renal fibrosis and identifying new therapeutic modalities have great significance for delaying CKD progression.

Endothelial cell dysfunction is a central pathophysiological mechanism that contributes to renal fibrosis, mainly via endothelial-mesenchymal transition and apoptosis (10, 11). The importance of endothelial cells and pericytes as a major source of renal collagen-producing cells following UUO has only recently been recognized, which shifts attention from the tubular epithelial cell to the renal vasculature as a focus for renal fibrotic injury (12). In addition, endothelial dysfunction promotes vascular permeability and leukocyte recruitment/adhesion, leading to further changes in renal perfusion and O_2_ delivery and thus to inflammation (13, 14). Importantly, dietary arginine supplementation improved renal fibrosis, apoptosis, and macrophage infiltration after UUO (15) by increasing the expression of endothelial NOS3 and enhancing arginine availability for NOS3 activity to nitric oxide (NO) in lieu of producing superoxide generator (16). Therefore, targeting therapeutic interventions to promote endothelial/epithelial function may be an effective strategy to reduce renal fibrosis.

Dramatic alterations in arginine metabolism occur in endothelial injury (17–19) due to changes in the activity and/or expression of NO synthases (NOS) and arginases. Arginase catalyzes hydrolysis of L-arginine to L-ornithine and urea and thus competes with NOS for the common substrate L-arginine (20). Depending on the stimulus, either or both arginases may be expressed and induced in macrophages, endothelial cells, and other cell types (21, 22). Arginase-2 (*Arg2*) is constitutively expressed and also inducible in kidney cells, such as endothelial cells (17–19) and tubular epithelial cells (23–25). Arg2 is localized in the mitochondria, with the highest expression in the kidney (21, 23). We showed that arginase inhibition or deficiency not only prevents the development, but also the progression of diabetic nephropathy in animal models of diabetes (25, 26) via an NOS3-dependent mechanism (27). However, the role of arginases in the pathogenesis of renal fibrosis is not known.

In the current study, we tested the hypothesis that arginases are a critical determinant of renal fibrosis. Toward this goal, we used several approaches such as arginase inhibition in conjunction with full-body Arg2-deficient mice and cell-specific Arg2 knockout in renal endothelial and proximal tubular cells in a well-established UUO fibrosis model. Our results indicate that targeting endothelial Arg2 activity or expression may be a novel therapeutic intervention to prevent kidney fibrosis and to reduce the incidence of kidney failure associated with CKD.

## Results

### Increased arginase expression and activity in renal UUO fibrosis model.

**Figure 1:**
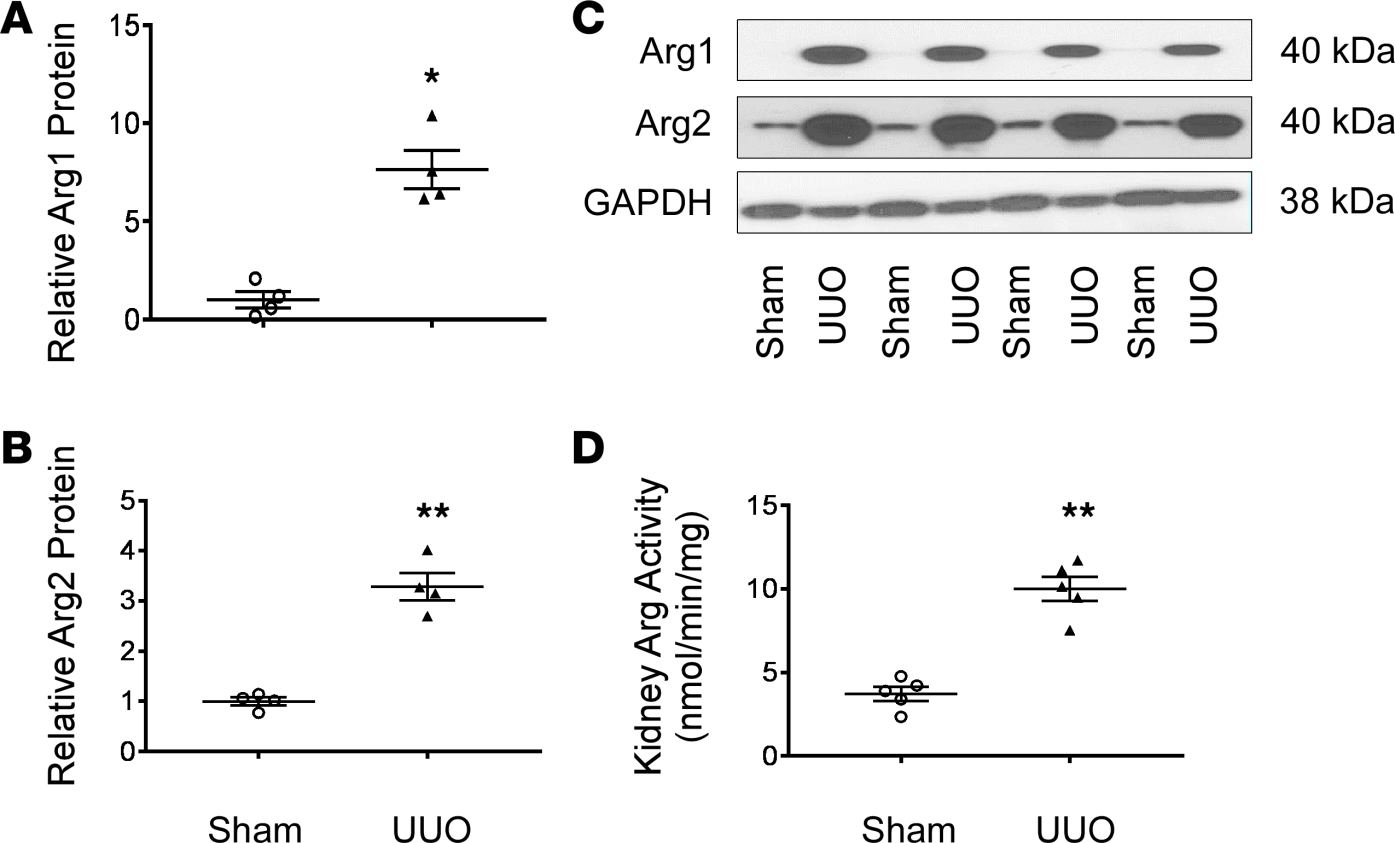
Arginases expression and activity increased in the UUO fibrosis model. The left ureter was ligated and kidneys were collected after 7 days. Arg1 (**A** and **C**) and Arg2 (**B** and **C**) protein levels were determined using Western blot (*n* = 4 each group). Kidney arginase activity was determined as described in the Methods (**D**) (*n* = 5 each group). Values are shown as mean ± SEM. *P < 0.05, **P < 0.01 compared with sham-operated mice using unpaired *t* test.

Male WT mice were subjected to UUO for 7 days, and then kidney tissues were removed and analyzed. Arg1 mRNA and protein expressions were virtually undetectable in sham-operated mice (Figure 1, A and C) but were increased in the obstructed kidney. Whole-kidney Arg2 mRNA and protein expression increased significantly after UUO (Figure 1, B and C), paralleling the increase in kidney arginase activity (Figure 1D) compared with sham-operated mice. This raises the possibility of an effect of *Arg1* and/or *Arg2* to mediate renal fibrosis.

### Inhibition of arginase reduces kidney fibrosis and fibrotic markers after renal UUO.

**Figure 2:**
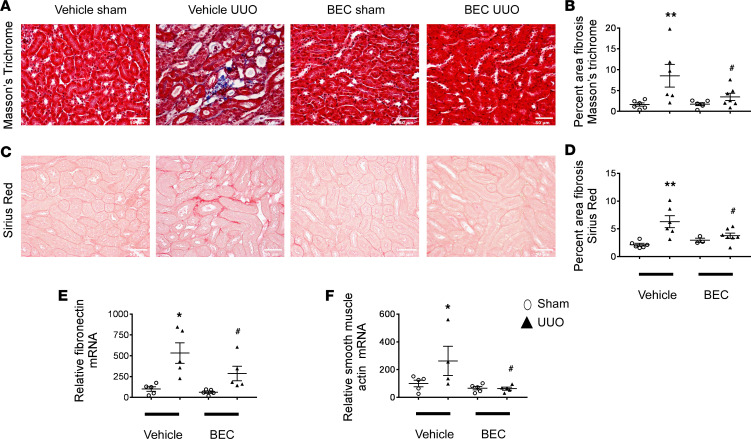
Arginase inhibition reduces kidney fibrosis and fibrotic markers following UUO. Representative images and quantitation of Masson’s trichrome– (**A** and **B**) and Sirius red–stained (**C** and **D**) left kidney sections. RT-PCR analysis of left kidneys from indicated mice subjected to UUO with or without BEC treatment for fibronectin (**E**) or smooth muscle actin (**F**). Values are shown as mean ± SEM. *P < 0.05, **P < 0.001 compared with vehicle sham; ^#^*P* < 0.05 compared with vehicle UUO using 1-way ANOVA (*n* = 3–7 each group). Scale bar: 50 μm (**A** and **C**).

WT mice were subjected to UUO and treated with either vehicle or the nonselective arginase inhibitor S-(2-boronoethyl)-L-cysteine (BEC) (2.3 mg/kg/d) via osmotic minipump for 7 days beginning at the time of surgery. Vehicle-treated UUO mice displayed an increase in the percentage of fibrosis (using ImageJ) in Masson’s trichrome– (Figure 2, A and B) and Sirius red–stained (Figure 2, C and D) sections 7 days after UUO injury, which was reduced by treatment with the arginase inhibitor BEC. Similarly, real-time PCR analysis show marked increases in kidney fibronectin (Figure 2E) and smooth muscle actin (Figure 2F) following UUO. Inhibition of arginases by BEC significantly blunted the increase in these fibrosis markers.

### Deficiency of Arg2 reduces kidney fibrosis and fibrotic markers after renal UUO.

**Figure 3:**
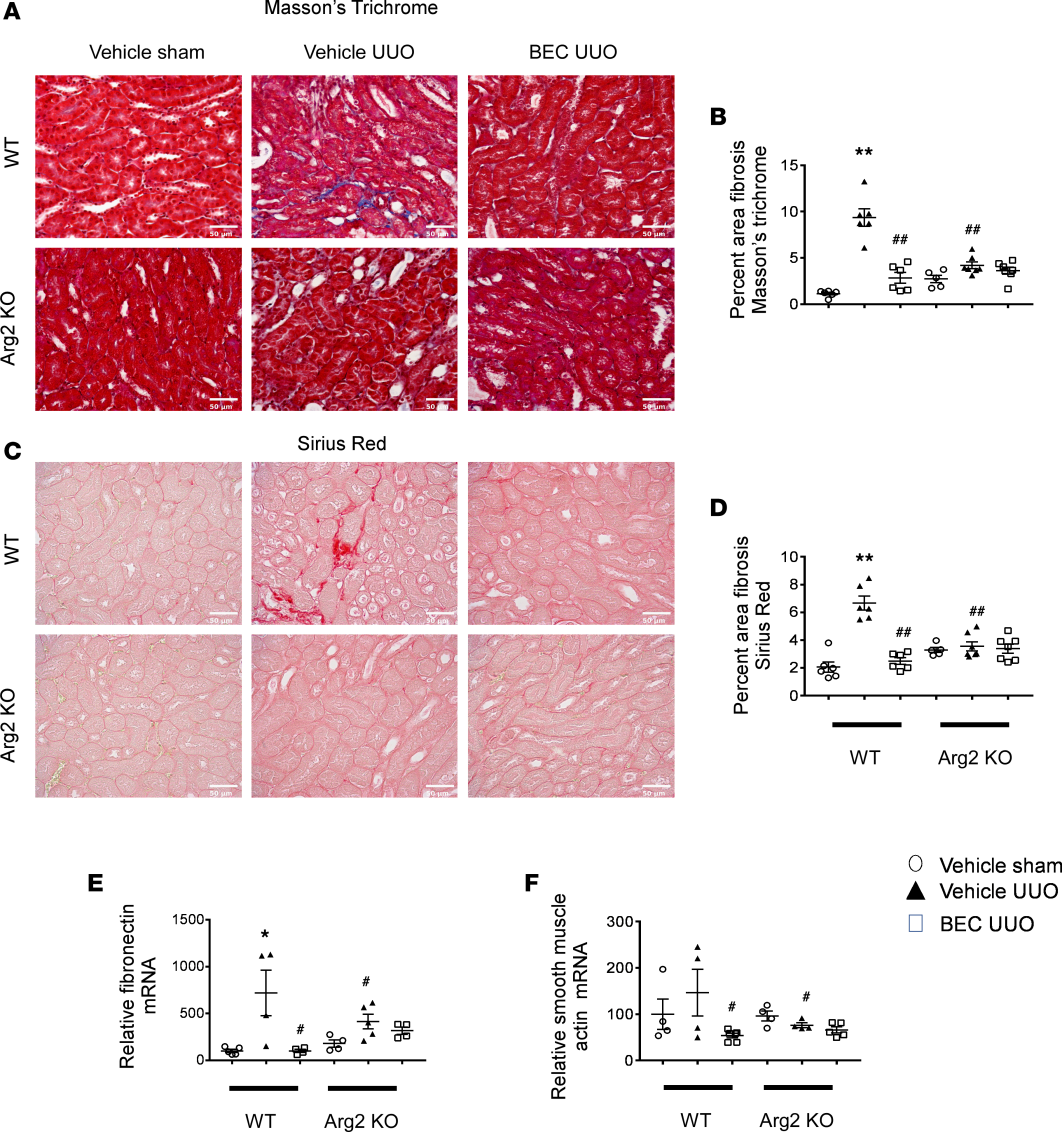
*Arg2* deficiency or arginase inhibition reduces kidney fibrosis and fibrotic markers following UUO. Representative images and quantitation of Masson’s trichrome– (**A** and **B**) and Sirius red–stained (**C** and **D**) left kidney sections. RT-PCR analysis of left kidneys from indicated mice for fibronectin (**E**) or smooth muscle actin (**F**). Values are shown as mean ± SEM. *P < 0.05, **P < 0.01 compared with WT sham; ^#^*P* < 0.05, ^##^*P* < 0.01 compared with WT UUO using 1-way ANOVA (*n* = 4–7 each group). Scale bar: 50 μm (**A** and **C**).

WT and *Arg2^–/–^* mice were subjected to UUO and treated with either vehicle or BEC via osmotic pump for 7 days. Whereas WT mice displayed an increase in the percentage of fibrosis in Masson’s trichrome– (Figure 3, A and B) and Sirius red–stained (Figure 3, C and D) sections 7 days after UUO injury, deletion of *Arg2* or inhibition of arginase in WT mice reduced the level of fibrosis after UUO. Similarly, real-time PCR analysis show marked reduction in whole-kidney fibronectin (Figure 3E) and smooth muscle actin (Figure 3F) after deletion of *Arg2* or inhibition of arginase in WT mice.

### Deficiency of Arg2 does not reduce kidney macrophage infiltration after renal UUO.

**Figure 4:**
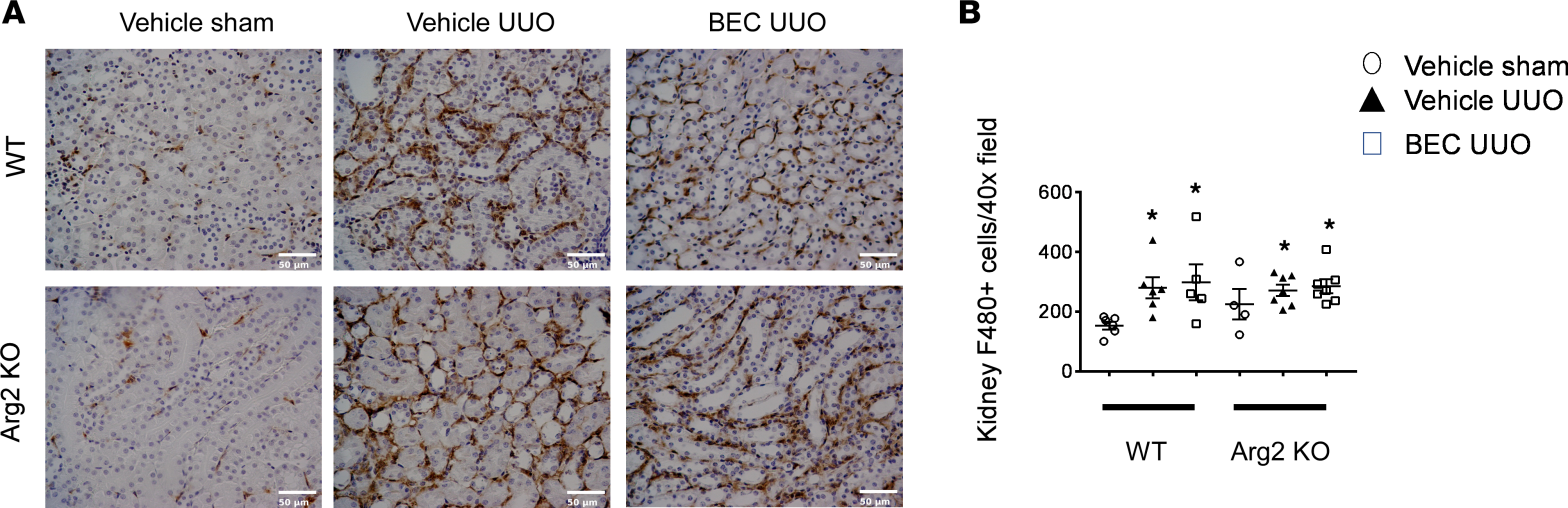
*Arg2* deficiency or arginase inhibition does not reduce kidney macrophage infiltration following UUO. Representative images (**A**) and quantitation of F4/80 stained left kidney sections (**B**). Values are shown as mean ± SEM. *P < 0.05 compared with WT sham-operated mice using 1-way ANOVA (*n* = 4–8 each group). Scale bar: 50 μm.

Both WT and *Arg2^–/–^* mice showed a marked increase in infiltrating kidney macrophages following UUO (Figure 4) compared with that in sham controls. Interestingly, BEC treatment did not reduce macrophage infiltration after UUO in either WT or *Arg2^–/–^* mice.

### Selective deficiency of Arg2 in endothelial cells, but not proximal tubular cells, reduces kidney fibrosis and fibrotic markers after renal UUO.

**Figure 5:**
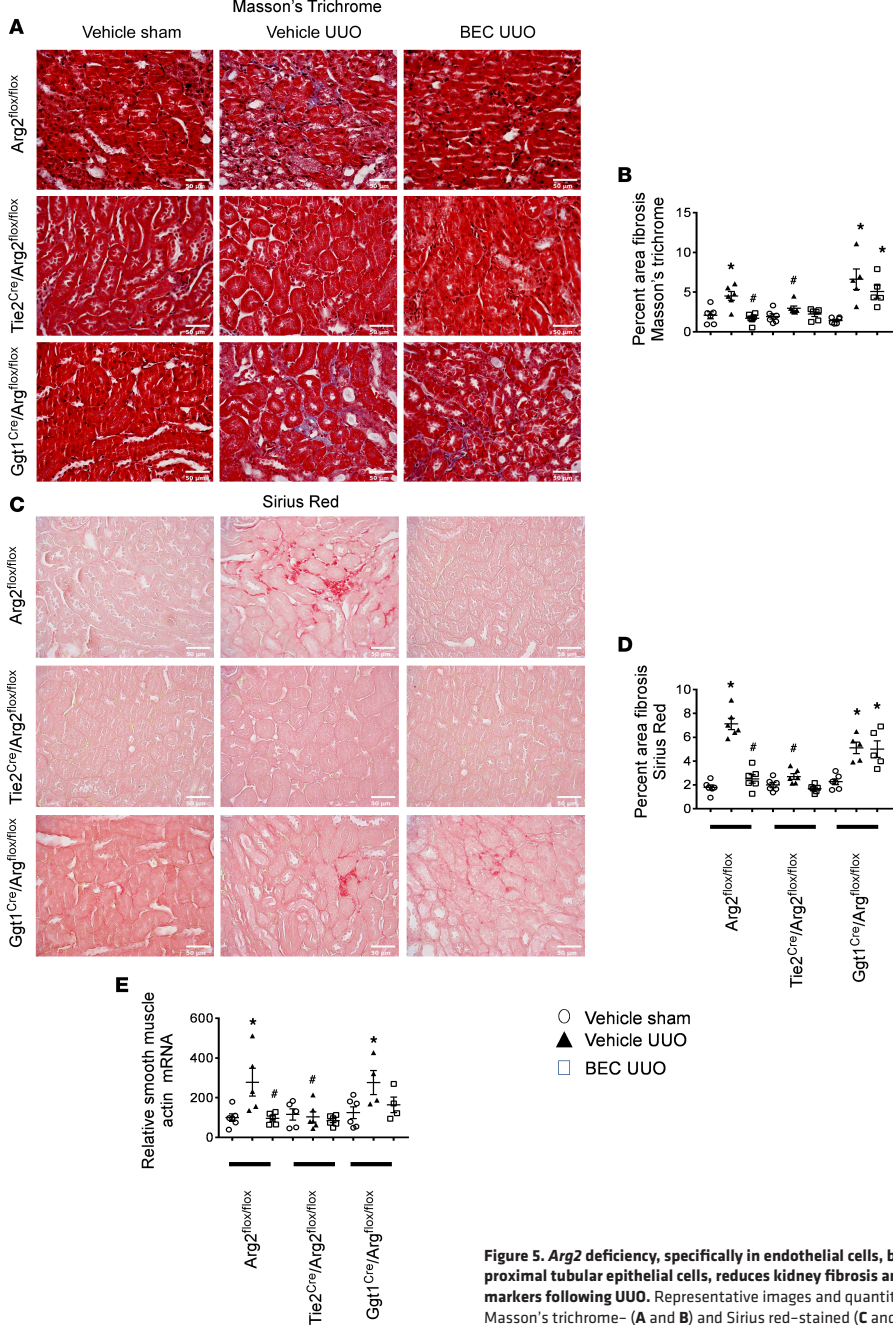
*Arg2* deficiency, specifically in endothelial cells, but not proximal tubular epithelial cells, reduces kidney fibrosis and fibrotic markers following UUO. Representative images and quantitation of Masson’s trichrome– (**A** and **B**) and Sirius red–stained (**C** and **D**) left kidney sections. RT-PCR analysis of left kidneys from indicated mice for smooth muscle actin (**E**). Values are shown as mean ± SEM. *P < 0.05 compared with *Arg2^fl/fl^* sham; ^#^*P* < 0.05 compared with *Arg2^fl/fl^* UUO using 1-way ANOVA (*n* = 5–9 each group). Scale bar: 50 μm (**A** and **C**).

We next sought to determine the effects of cell-specific arginase deletion in proximal tubular and endothelial cells using *Ggt1^Cre^/Arg2^fl/fl^* and *Tie2^Cre^/Arg2^fl/fl^* mice, respectively, following UUO. We first generated and confirmed *Arg2^fl/fl^* mice with 2 LoxP site (Supplemental Figure 1; supplemental material available online with this article; https://doi.org/10.1172/jci.insight.142187DS1). *Arg2^fl/fl^* mice were crossed with commercially available *Ggt1^Cre^* and *Tie2^Cre^* mice to obtain *Arg2*-specific deletion in proximal tubular and endothelial cells (Supplemental Figure 2A). Tubular epithelial and endothelial cells were isolated from kidneys to confirm *Arg2* deletion from *Ggt1^Cre^/Arg2^fl/fl^* (Supplemental Figure 2B) and *Tie2^Cre^/Arg2^fl/fl^* (Supplemental Figure 2C) mice, respectively. As expected, *Arg2^fl/fl^* mice (controls) displayed an increase in the percentage of fibrosis in Masson’s trichrome– (Figure 5, A and B) and Sirius red–stained (Figure 5, C and D) sections 7 days after UUO injury. In contrast, deletion of *Arg2* specifically in endothelial cells (*Tie2^Cre^/Arg2^fl/fl^*) reduced the level of fibrosis after UUO. Interestingly, deletion of *Arg2* specifically in proximal tubular cells (*Ggt1^Cre^/Arg2^fl/fl^*) failed to reduce renal fibrosis and was similar to vehicle-treated UUO in *Arg2^fl/fl^* mice. Similarly, real-time PCR analysis of *Arg2^fl/fl^* mice (controls) showed marked increases in whole-kidney smooth muscle actin (Figure 5E) following UUO. In contrast, deletion of *Arg2* specifically in endothelial cells (*Tie2^Cre^/Arg2^fl/fl^*), but not in proximal tubular cells (*Ggt1^Cre^/Arg2^fl/fl^*), reduced whole-kidney α-smooth muscle actin after UUO.

### Inhibition of arginases restores kidney NO and mitochondrial function after renal UUO.

**Figure 6:**
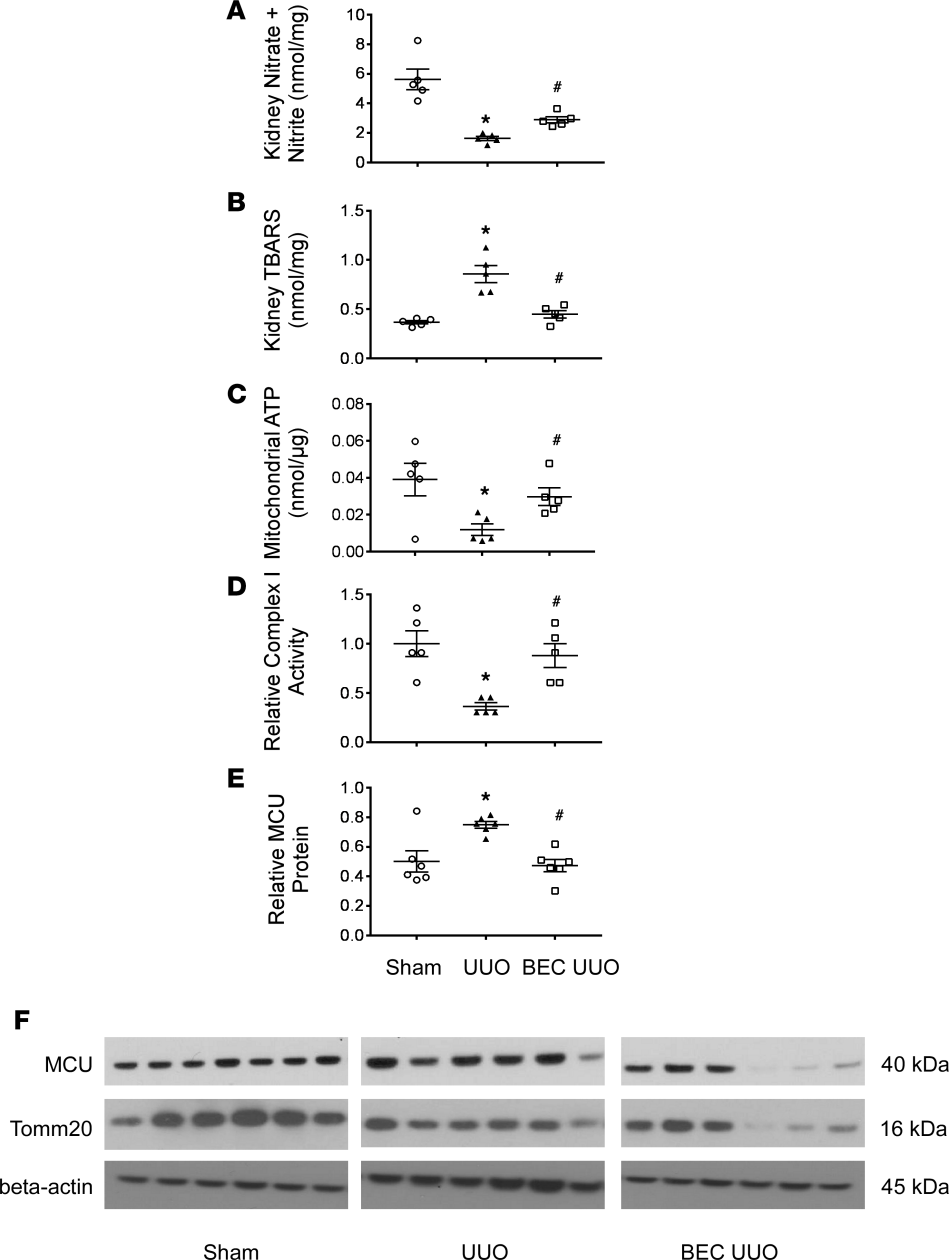
Arginase inhibition restored kidney NO and restored mitochondrial functions following UUO. (**A**) Levels of nitrate and nitrite were determined from whole left kidney tissue 7 days after UUO. (**B**) TBARS assay following UUO in whole-kidney tissue. (**C**) Levels of mitochondrial ATP from indicated left kidney tissue. (**D**) Complex I activity levels from indicated left kidney tissue. (**E** and **F**) Protein was isolated form left kidney sections for WT mice subjected to UUO for 7 days. Western blotting for MCU normalized to Tomm20 as a mitochondrial marker, while β actin show a comparable protein loading. Values are shown as mean ± SEM. *P < 0.05 compared with vehicle sham; ^#^*P* < 0.05 compared with vehicle UUO using 1-way ANOVA (*n* = 5–6 each group).

Renal UUO reduced total kidney nitrate and nitrite end products (Figure 6A) and increased kidney thiobarbituric acid reactive substances (TBARS) as an indicator for oxidative stress (Figure 6B), whereas arginase inhibition significantly restored total kidney nitrate and nitrite end products and TBARS levels after UUO. In addition, mice subjected to UUO had a marked decrease in kidney levels of mitochondrial ATP (Figure 6C) and complex I activity (Figure 6D), and this was reversed with BEC treatment. In contrast, vehicle-treated UUO mice had a significant increase in kidney mitochondrial Ca^2+^ uniporter complex (MCU) protein expression compared with sham-operated mice after 7 days of UUO (Figure 6E). In UUO mice, treatment with an arginase inhibitor restored MCU complex levels to normal.

## Discussion

Fibrosis is the final common pathway in the pathophysiology of most forms of CKD (1–3). Currently, no available treatment modalities exist for renal fibrosis. Arginases have a well-established role to alter endothelial function in cardiovascular diseases (17–19), yet their role in renal fibrosis has not previously been determined. To examine the direct role of arginases in renal fibrosis along with its cellular target and mechanism of action, we used several approaches in genetically altered mice in a well-established UUO fibrosis model. First, we showed increased Arg2 expression and arginase activity following renal fibrosis. Second, we showed that pharmacological blockade or genetic deficiency of *Arg2* mediates renal tissue protection following renal fibrosis, as reflected by preservation of kidney interstitial fibrosis and fibrotic markers. Our results confirm those of a previous report showing that arginase inhibition protects the kidney from structural damage in the 5/6 renal mass ablation/infarction model of CKD (28). Third, we showed using our newly developed *Arg2^fl/fl^* mice that selective deletion of *Arg2* specifically in endothelial cells, but not in proximal tubular epithelial cells, reduced the level of fibrosis after UUO. Our isolated endothelial and proximal tubule cells showed high but not total Arg2 knockdown, but the deficiency was enough to be considered relevant for our studies and conclusions. Fourth, we investigated the possible mechanism(s) of action of *Arg2* and found that *Arg2*’s effects could be mediated via restoring NO, oxidative stress, and mitochondrial function following UUO fibrosis model. Our study, however, reflects the short-term nature of UUO and that additional longer-term studies are needed. Previous reports showed that treatment with arginase inhibitors reduced fibrosis and collagen deposition in aorta (29, 30), heart (30), lung (31–33), intestine (34), and peritoneal membrane (35). Since the liver has a very high amount of Arg1, enormous amounts of an inhibitor would be required to inhibit liver arginase sufficiently for any inhibition of the urea cycle to become apparent. Importantly, the reactions of the urea cycle are not diffusion controlled but are tightly coupled, such that arginine generated within the urea cycle and used by arginase does not exchange with the free arginine pool within the cell (36). Consequently, a competitive arginase inhibitor will not exchange with arginine generated within the urea cycle and thus should have little to no effect on the urea cycle. Furthermore, competitive arginase inhibitors have been used in many animal studies, and hyperammonemia or other adverse effects have not been reported in any of them. Our findings, therefore, reveal an important role for *Arg2* in the pathogenesis of renal fibrosis and provide evidence for arginase inhibition as a potential new therapeutic modality for treating patients with CKD.

Arg2 is highly expressed in kidney cells, such as endothelial cells (17–19) and tubular epithelial cells (23–25). Unlike Arg2, Arg1 is undetectable at the protein level under normal conditions (our data and ref. 25), yet both isoforms were elevated following renal fibrosis, indicating a possible role of *Arg2* and/or *Arg1* to mediate renal tissue injury following UUO. *Arg1* and *Arg2* are encoded by different genes, differ with regard to their tissue distribution and subcellular localization, and are independently regulated (21, 22). The fact that Arg1 protein was elevated in renal fibrosis suggests a possible contribution of *Arg1* to the development and progression of renal fibrosis, possibly via its expression in infiltrating macrophages. Additional experiments are needed to explore this possibility. The fact that pharmacologic blockade or genetic deficiency of *Arg2* reduced renal fibrosis indicates that *Arg2* is the primary target for arginase inhibition. Increased expression of Arg1 and Arg2 in endothelial cells stimulated conversion of arginine to proline (37), suggesting that this may be one mechanism whereby elevated arginase contributes to increased fibrosis in UUO.

Endothelial dysfunction, characterized by reduced bioavailability of NO and increased oxidative stress, is a hallmark of renal fibrosis, mainly via capillary loss and consequent renal ischemia and hypoxia (10, 11). NO is produced from arginine by NOS. Under conditions of low arginine availability or hypoxia, endothelial NOS (NOS3) is uncoupled, producing ROS and oxidative stress in lieu of NO (38, 39). Our data show that the protective effect of arginase inhibition to reduce renal fibrosis could be mediated by restoring NO levels and oxidative stress. The mechanism by which arginase inhibition reduces kidney oxidative stress could be due to decreased immune response or increased availability of arginine to NOS3. Endothelial cells have been recently recognized as a pivotal target for renal fibrosis (12). Similarly, dysfunction and loss of tubular epithelial cells play a central role in renal fibrosis. Renal tubules are very sensitive to oxygen deprivation or nephrotoxic substances (40, 41), likely due to reduced ATP production and/or function and generation of ROS (42). Renal tubular cells can also produce a number of proinflammatory cytokines, including TNFA, IL6, and TGFB, and chemokines, such as RANTES, MCP1, ENA78, GroA, and IL8 (43, 44). Therefore, targeting therapeutic interventions to promote endothelial/epithelial function may be an effective strategy to reduce renal fibrosis. Toward this goal, we further defined the contribution of proximal tubule and endothelial cell arginases in kidney injury using tissue-specific deletion of *Arg2* in proximal tubule and endothelial cells. Our data show that selective deletion of *Arg2* specifically in endothelial cells blunted the increase in renal fibrosis after UUO, indicating a possible direct role of endothelial cell *Arg2* in the pathogenesis of renal fibrosis. It is important to note that Tie2^cre^ may result in Arg2 being knocked out both in endothelial and immune cells (45). Arg2 is expressed in bone marrow–derived monocyte/macrophages, and activation of M1 macrophages by LPS exclusively induces Arg2 but not Arg1 expression in murine and human macrophages (46). Conversely, silencing Arg2 expression in human monocyte/macrophage cell lines or macrophages from Arg2^–/–^ mice decreases proinflammatory cytokine levels (46). We, however, speculate that endothelial cells are the primary trigger for *Arg2* effect. This conclusion is based on our finding demonstrating increased macrophage infiltration after UUO in both Arg2 WT and Arg2-knockout mice compared with sham-operated mice. Arginase inhibition failed to reduce the elevated macrophage infiltration after UUO, indicating that the protective effect of arginase deficiency and arginase inhibition in UUO model is independent of macrophages infiltration. Interestingly, while deletion of *Arg2* specifically in proximal tubules failed to reduce renal fibrosis, indicating that proximal tubular epithelial cell *Arg2* is not important to mediate renal tissue injury, arginase inhibition also failed to reduce renal tissue fibrosis in *Ggt1^Cre^/Arg2^fl/fl^* mice after UUO, indicating that part of the effect of arginase inhibition could be mediated through *Arg1* and/or nonproximal tubular epithelial cells. Although in vivo inhibition of arginases improved high blood pressure (47), our data showed no differences in blood pressure between *Tie2^Cre^/Arg2^fl/fl^* and *Ggt1^Cre^/Arg2^fl/fl^* mice compared with *Arg2^fl/fl^* mice (Supplemental Table 1). Similarly, our previous reports (25–27, 48) showed that the beneficial effects of arginase inhibition were not due to reductions in blood pressure in diabetic nephropathy model.

Mitochondrial dysfunction has emerged as a new therapeutic target in renal injury (49–58). In the kidney, proximal tubules and endothelial cells are especially vulnerable to mitochondrial dysfunction (53, 59–63) and contribute to oxidative stress, persistent energy depletion, impairment of energy-dependent repair mechanisms, and cell death in kidney injury (59, 60). Mitochondrial Ca^2+^ (_m_Ca^2+^) uptake is driven by the electrochemical gradient across the inner mitochondrial membrane and facilitated by the highly selective _m_Ca^2+^ uniporter (MCU) (64–66). The MCU complex comprises multiple functional domains with the MCU as the central pore-forming subunit (67). MCU is a multimeric complex that mediates the rapid uptake of cytosolic Ca^2+^ from intracellular store release (68–76). We have shown an endothelial cell mechanotransduction-mediated regulation of MCU activity in the context of vascular physiology (77). MCU has been shown to be dysregulated in hypoxia and ischemia/reperfusion injury; additionally, it has been shown that restoration of MCU function prevented mitochondrial dysfunction and cell death (78). Importantly, *Arg2* is localized in the mitochondria, with the highest expression in the kidney and endothelial cells (21, 24). However, the role of *Arg2* in kidney mitochondrial dysfunction and/or MCU complex in renal fibrosis has not been investigated. Our data show that arginase inhibition restored the reduction in kidney mitochondrial ATP, complex 1 activity, and MCU complex. To our knowledge this is the first study linking MCU with renal fibrosis. Our results, therefore could support a role for *Arg2* in mitochondrial biogenesis following renal fibrosis. Additional studies are needed to confirm a cause-effect relationship.

In conclusion, our study demonstrates for the first time to our knowledge that *Arg2* plays an essential role in the development of renal fibrosis, mainly by targeting Arg2 expressed in endothelial cells. The mechanism of renal tissue protection using arginase inhibition in renal fibrosis could be mediated by its effect on endothelial cells and mitochondrial function. Findings of our study may ultimately result in novel therapeutic interventions designed to attenuate arginase activity or signaling that regulates Arg2 expression in the treatment of renal fibrosis.

## Methods

### UUO mouse model.

Experiments were conducted in male 8-week-old C57BL/6J and *Arg2^–/–^* mice (The Jackson Laboratory). For the UUO model, mice were anesthetized, and a midline incision was made as described previously (79). The left ureter was tied off twice with a nonabsorbable silk surgical suture. Osmotic pumps (Durect Corporation) containing either PBS (vehicle) or BEC (2.3 mg/kg/d; Cayman Chemical) were implanted subcutaneously at the time of surgery as we described previously (25, 26). Sham surgeries were conducted in a similar manner, except the ureter was not tied off. These sham-operated mice were used as controls throughout the study. Following surgery mice were placed in a warm cage and observed until they recovered. Kidney tissue was collected 7 days after UUO surgery. Mouse systolic blood pressure were recorded using the CODA Noninvasive Blood Pressure system (Kent Scientific Corporation) as we described previously (25). Mice were acclimated for 10 minutes at 26°C before readings began. Readings were taken at the same time of day for all groups to prevent any diurnal variations.

### Generation of Arg2-specific knockout mice.

*Arg2^fl/fl^* mice were developed by Cyagen. The mouse *Arg2* gene (GenBank accession NM_009705.3), encoded by 8 exons, is located on mouse chromosome 12. Exon 3 was selected as conditional knockout region, as its deletion will result in the loss of function of the Arg2 gene. To engineer the targeting vector, homology arms and conditional KO (cKO) region were generated by PCR using BAC clone RP24-73D13 and RP23-110C12 from the C57BL/6J library as a template. In the targeting vector, the Neo cassette was flanked by Frt sites, and cKO region was flanked by LoxP sites (Supplemental Figure 1). Several pups with the desired KO construct were identified by multiple PCR screenings. Homozygous *Arg2^fl/fl^* mice were crossed with commercially available *Ggt1^Cre^* and *Tie2^Cre^* mice (The Jackson Laboratory, catalog 008863 and 12841, respectively) to obtain *Arg2*-specific knockout in proximal tubular and endothelial cells (Supplemental Figure 2A).

### Kidney-derived cell isolation.

Proximal tubular epithelial and endothelial cells were isolated from kidneys to confirm *Arg2* deletion from *Ggt1^Cre^/Arg2^fl/fl^* (Supplemental Figure 2B) and *Tie2^Cre^/Arg2^fl/fl^* (Supplemental Figure 2C) mice, respectively, as described previously (80–83). For endothelial cells, kidneys were removed on ice, stripped of their outer capsules, and cortexes were minced before being placed in 1 mg/mL collagenase (MilliporeSigma) solution at 37°C with shaking for 45 minutes. Tissues were then passed through a 70 μM filter twice, centrifuged at 400*g* for 5 minutes at 4°C, and endothelial cells were separated using CD31 magnetic beads (Miltenyi Biotec). Cells were then grown on coated plates with RPMI-1640 (Thermo Scientific) plus 10% fetal bovine serum for 7 to 10 days before being used for experiments. For proximal tubules isolation, we used a modified method as described previously (84). Specifically, mice were perfused with 0.5% iron III oxide (MilliporeSigma), and then kidney cortexes were isolated and minced before being digested in 1 mg/mL collagenase at 37°C with shaking for 30 minutes and filtered with 250 and 70 μM cell strainers. Supernatants were then centrifuged at 50 *g* for 5 minutes, and then the iron-coated pellet was grown on plates precoated with 20 mM acetic acid and 5 μg collagen type 1 (Thermo Scientific) in RPMI-1640 plus 10% fetal bovine serum overnight. Media were then collected next day and centrifuged at 50*g* for 4 minutes, and then pellets suspended in fresh growth media and returned to the original plate. Proximal tubule cells were grown to confluence for 4 to 7 days, then used for experiments.

### Western blotting.

Kidney tissue was homogenized in RIPA buffer containing 0.1% Triton X-100 supplemented with protease inhibitors (Roche Diagnostics) and cleared by centrifugation at 10,000*g* for 10 minutes at 4°C. Cells were scraped from vessels using cell scrapers and centrifuged at 10,000*g* for 10 minutes at 4°C, and supernatant was collected. Protein concentration was determined by Bicinchoninic Acid assay (Thermo Scientific). Fifty μg of kidney or cell lysate was separated on a 4%–12% Bis-Tris gel (Life Technologies) and transferred onto PVDF membranes before blocking with 5% dry milk. Western blots were performed using the following antibodies: arginase-1 (catalog sc-271430, 0.4 μg/mL, Santa Cruz Biotechnology), Arg2 (catalog sc-374420, 0.4 μg/mL, Santa Cruz Biotechnology), Tomm20 (catalog 42406, 1:1000, Cell Signaling), MCU (catalog 14997, 1:1000, Cell Signaling), GAPDH (catalog 5174, 1:2000, Cell Signaling), and β-actin (catalog 4970, 1:1000, Cell Signaling) antibodies. Western blots were quantitated using ImageJ software (NIH) and normalized to loading control protein expression.

### RNA isolation and real-time PCR.

RNA was isolated by Trizol extraction from whole-kidney sections and reverse transcribed to cDNA using the Bio-Rad iScript cDNA synthesis kit. A 1:50 dilution of the cDNA was prepared and used for real-time PCR analysis using SYBR Green Master Mix (Thermo Scientific), as previously described (25). Primers and sequences used are as follows: fibronectin (forward, GTCCTGTGGGAGGGGTGTTTGA; reverse, TGCTTTCTTTTGCCATCTGACCTG), smooth muscle actin (forward, CTGCCGTTTTCCCCCTTCCTCT; reverse, TTGCTTCCTCCTCCTTTGG), and β-actin (forward, DCTGTTTGTGTAAGGTAAGGTGTCG; reverse, GAGGGGGTTGAGGTGTTGAGG). Relative levels of mRNA were calculated as previously described (25, 85).

### Histology and immunohistochemistry.

Sections of left kidney tissues were fixed in 10% neutral-buffered formalin and embedded in paraffin, and 3 μm sections were cut. Sections were then stained with Masson’s trichrome or Sirius red. To determine the percentage area of fibrosis using Masson’s trichrome or Sirius red, images were obtained at ×10, saved as jpeg files, and analyzed in ImageJ. F4/80 macrophage staining was performed on sections similar to methods previously described (86) using F4/80 antibody (catalog sc-59171, 1:400, Santa Cruz Biotechnology). Images were taken on a Nikon Eclipse E600 scope using a Nikon Digital Camera DYM1200.

### Measurement of total nitrate and nitrite.

Fresh kidney homogenates were prepared in 1× PBS, pH 7.4, and clarified supernatants were used to determine protein concentration and total nitrate/nitrite using the Nitrate/Nitrite Colorimetric Assay Kit (Cayman Chemical) as previously described (26, 87, 88).

### TBARS assay.

Proteins were precipitated from kidney homogenates using 10% trichloroacetic acid, and subsequent supernatant was used for TBARS assay as described previously (26, 89).

### Arginase activity assay.

Kidney lysates were assessed for arginase activity as previously described (25, 87).

### Mitochondria isolation and function.

Mitochondria were isolated from kidney tissue using a mitochondria isolation kit (Thermo Scientific). ATP levels were assessed using a luciferase-based assay (Promega) following the manufacturer’s instructions. Complex I activity was measured as previously described by Birch-Machin and Turnbull (90).

### Statistics.

Comparisons between groups were conducted using GraphPad Prism software (version 7.04). Results are expressed as mean ± SEM. Unpaired 2-tailed *t* test was used for comparison between 2 groups. One-way ANOVA was used to compare significance between more than 2 groups. A *P* value of less than 0.05 was considered statistically significant.

### Study approval.

All animal studies were approved by the Penn State University College of Medicine (Hershey, Pennsylvania, USA) and University of Texas Health Science Center at San Antonio Institutional Animal Care and Use Committees.

## Author contributions

ASA designed research studies. MDW, KS, WWW, and SM conducted experiments, acquired data, and analyzed data. ASA and MM analyzed data. MDW and ASA wrote the manuscript. MDW, KS, WWW, SM, MM, WBR, and ASA reviewed and edited the manuscript. MM and WBR provided critical advice.

## Supplementary Material

Supplemental data

## Version 1 09/17/2020

In-Press Preview

## Version 2 10/02/2020

Electronic publication
