# Supplementary material for: Selective inhibition of arginase-2 in endothelial cells but not proximal tubules reduces renal fibrosis
Source: JCI Insight. 2020 Oct 2;5(19):e142187. doi: 10.1172/jci.insight.142187 (PMC7566719; doi:10.1172/jci.insight.142187)
Supplement: Supplemental data [file jciinsight-5-142187-s163.pdf]

Supplemental Table 1: Blood pressure measurement on transgenic mice.

Mouse type	Systolic blood pressure (mmHg)
$Arg2^{flox/flox}$	104.99 ± 15.95
$Ggt1^{Cre}/Arg2^{flox/flox}$	89.54 ± 4.02
$Arg2^{flox/flox}$	116.83 ± 3.15
$Tie2^{Cre}/Arg2^{flox/flox}$	117.14 ± 4.02

Systolic blood pressure was measured on $Arg2^{flox/flox}$, $Ggt1^{Cre}/Arg2^{flox/flox}$, and $Tie2^{Cre}/Arg2^{flox/flox}$ mice. (n=4 each group). Values are means ± SEM, analyzed using unpaired t-test.

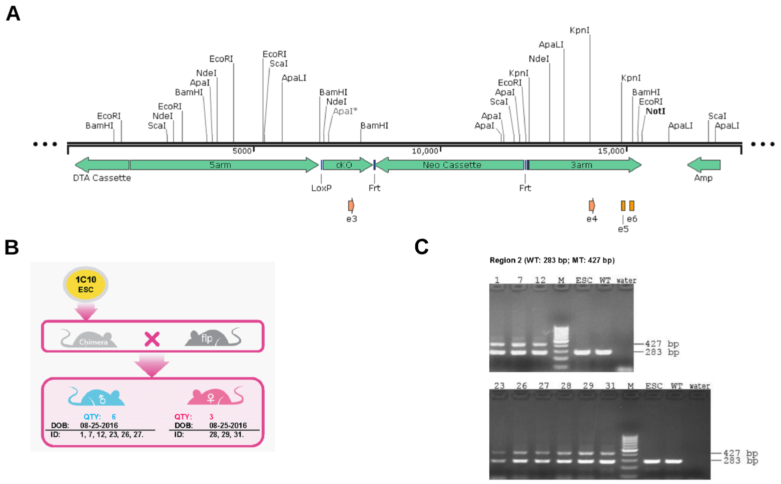

Supplemental Fig. 1: Generation of Arg2$^{flox/flox}$ mice. (A) Design for insertion of LoxP sites into Arg2 gene. (B) Embryonic stem (ES) cells were implanted into a chimeric mouse and crossed with flp delete mouse to get Arg2$^{flox/WT}$ offspring. (C) Arg2$^{flox/WT}$ was confirmed by PCR analysis of 2 flox region deletion sites at 427 (mutant) and 283 (WT) bp.

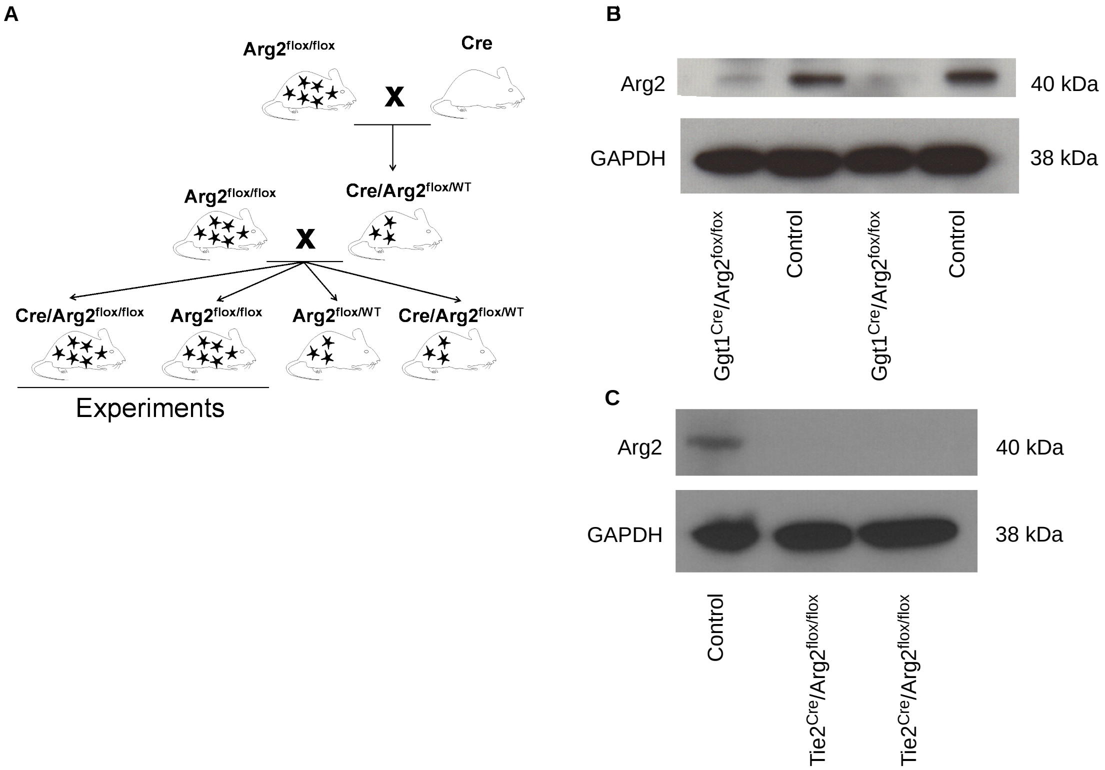

Supplemental Fig. 2: Generation and confirmation of Ggt1^{Cre}/Arg2$^{flox/flox}$ and Tie2^{Cre}/Arg2$^{flox/flox}$ mice. (A) Arg2$^{flox/flox}$ mice were cross-bred with Ggt1^{Cre} and Tie2^{Cre} mice for 2 generation to obtain Ggt1^{Cre}/Arg2$^{flox/flox}$ and Tie2^{Cre}/Arg2$^{flox/flox}$; respectively. (B-C) Western blot analysis of Arg2 expression in isolated proximal tubules and endothelial cells from Ggt1^{Cre}/Arg2$^{flox/flox}$ and Tie2^{Cre}/Arg2$^{flox/flox}$. Black stars represent the Arg2$^{flox/flox}$ status.
